# Hybrid luminescence materials assembled by [Ln(DPA)_3_]^3−^ and mesoporous host through ion-pairing interactions with high quantum efficiencies and long lifetimes

**DOI:** 10.1038/srep08385

**Published:** 2015-02-11

**Authors:** Qing-Feng Li, Dan Yue, Wei Lu, Xinlei Zhang, Chunyang Li, Zhenling Wang

**Affiliations:** 1 The Key Laboratory of Rare Earth Functional Materials and Applications, Zhoukou Normal University, Zhoukou 466001, P. R. China; 2 Department of Applied Physics and Materials Research Center, The Hong Kong Polytechnic University, Hong Kong, P. R. China

## Abstract

A kind of mesoporous hybrid luminescence material was assembled through the ion exchange method between [Ln(DPA)_3_]^3−^ and ionic liquid functionalized SBA-15. [Ln(DPA)_3_]^3−^ was successfully anchored onto positive-charge modified SBA-15 by the strong electrostatic interaction. In [Ln(DPA)_3_]^3−^, Ln^3+^ ions are in 9-fold coordination through six oxygen atoms of carboxyl groups and three nitrogen atoms of pyridine units, leaving no coordination site for water molecules. Therefore the hybrids possess prominent luminescent properties, SBA-15-IMI-Tb(DPA)_3_ and SBA-15-IMI-Eu(DPA)_3_ exhibit high quantum yield values of 63% and 79%, and long lifetimes values of 2.38 ms and 2.34 ms, respectively. Especially, SBA-15-IMI-Eu(DPA)_3_ presents a high color purity, and the red/orange intensity ratio is as high as 7.6. The excellent luminescence properties and ordered mesoporous structures give rise to many potential applications in optical and electronic areas.

Lanthanide complexes with excellent luminescence properties have attracted considerable attention due to their outstanding emission properties such as long luminescence lifetime, photostability, narrow and easily identifiable emission lines. These distinctive properties make them be suitable for application in lighting devices and biomedical analysis^1^,^2^. In such complexes, appropriate organic ligands such as β-diketones^3^,^4^,^5^, phenanthroline^6^ and heterocyclic carboxylic acid group^7^,^8^,^9^ usually act as antenna or sensitizers which could absorb the light energy and transfer the energy from the lowest triplet state energy level (T_1_) of these organic groups to the resonance level of the lanthanides. Furthermore, the organic ligands also shield the lanthanides from coordinated solvent molecules (such as water), and these solvent molecules can diminish the luminescence of the complexes because the high energy vibration of O-H will induce the non-radiative transition of lanthanides.

However, the pure lanthanide complexes are not suited for use in optical devices mainly due to their poor thermal stabilities and low mechanical strength. As a result, increasing attention has been focused on the design and synthesis of organic-inorganic hybrid materials by grafting of lanthanide complexes on polymer matrices^10^,^11^,^12^, silica-based materials^13^,^14^,^15^,^16^,^17^,^18^, and liquid crystals^19^,^20^, et al. For example, Reddy and coworkers reported a luminescence carbon material by bonding the visible sensitized Eu^3+^ β-diketonate complex to carboxyl functionalized multi-walled carbon nanotube^21^. Wang and coworkers prepared a hybrid luminescence material by linking Eu^3+^ or Tb^3+^ complexes with a new polydentate amide ligand to mesoporous silica nanospheres (MSNs) for the recognition of fluoride ions^22^. Lin and coworkers designed a paramagnetic hybrid material by grafting Gd^3+^ complex to the thiol-functionalized MSNs through a disulfide tether, which could act as a magnetic resonance imaging contrast agent^23^. Generally, the novel hybrids will exhibit superior physicochemical and optical properties, including high thermal stabilities and mechanical strength, long luminescence lifetimes, as well as promising application. Among all of the lanthanide hybrids, mesoporous silicas with unique properties (large surface area, uniform and adjustable pore sizes, as well as easy surface modification) are frequently used as the scaffolding materials due to their potential applications in sensing, drug delivery, biomedical imaging, etc. Up to now, lanthanide complexes have been incorporated into mesoporous silica matrices, such as MCM-41, SBA-15 by a covalent bond grafting technique or a non-covalent, assembling method^24^,^25^.

Ionic liquids functionalized mesoporous materials have drawn considerable attentions over the past decades, owing to theirs application in electrochemistry, catalysis and separation and adsorption^26^,^27^. Recently, Mesoporous silicas modified with alkylimidazolium quaternary ammonium salt were used as the novel anion exchange matrixs for preparation of the hybrid mesoporous luminescence materials^28^,^29^. A negatively charged lanthanide complexes could be non-covalently immobilized on mesoporous materials grafted with a positively charged group through strong electrostatic interactions. A series of luminescence materials have been synthesized through the incorporation of europium β-diketone complexes into functionalized mesoporous materials, which exhibit a bright red photoluminescence upon illumination with ultraviolet light. However, β-diketones are not the efficient ligands for sensitizing the luminescence of terbium ion, because the lowest triplet state energy level of the β-diketones can not match the emission energy level of terbium ion very well^30^.

Pyridine-2, 6-dicarboxylic acid (DPA) is an efficient tridentate N, O-chelating ligand for sensitization of terbium or europium ions luminescence. Three DPA ligands are able to coordinate with the lanthanide ion through six oxygens of carboxyl groups and three nitrogen atoms of pyridine units, resulting in the formation of [Ln(DPA)_3_]^3−^ (Ln: Tb, Eu)^31^,^32^,^33^,^34^. The coordination sphere of the lanthanide ion is saturated by the ligands, leaving no coordination site for water molecules. Therefore, [Ln(DPA)_3_]^3−^ exhibit excellent luminescence properties in aqueous solution^33^. Moreover, [Ln(DPA)_3_]^3−^ with three negative charges could be incorporated into the positive-charge modified mesoporous materials using an ion exchange method. In addition, the luminescence properties of the complexes formed by lanthanides and DPA derivatives can be adjustable by changing the substituents on the DPA or lanthanides. In this work, we propose a suitable and convenient method to assemble a novel mesoporous luminescence material (SBA-15-IMI-Ln(DPA)_3_, Ln = Tb or Eu). [Ln(DPA)_3_]^3−^ was incorporated into the alkylimidazolium quaternary ammonium salt functionalized mesoporous materials by ion-pairing interactions. The coordination feature of Ln^3+^ and well energy match between the energy levels of DPA and Ln^3+^ make the hybrids possess excellent luminescent properties such as high luminescent quantum yields, long lifetimes, high color purity and multi-color luminescence (red and green) under the excitation of a single wavelength ultraviolet light.

## Results

**Figure 1:**
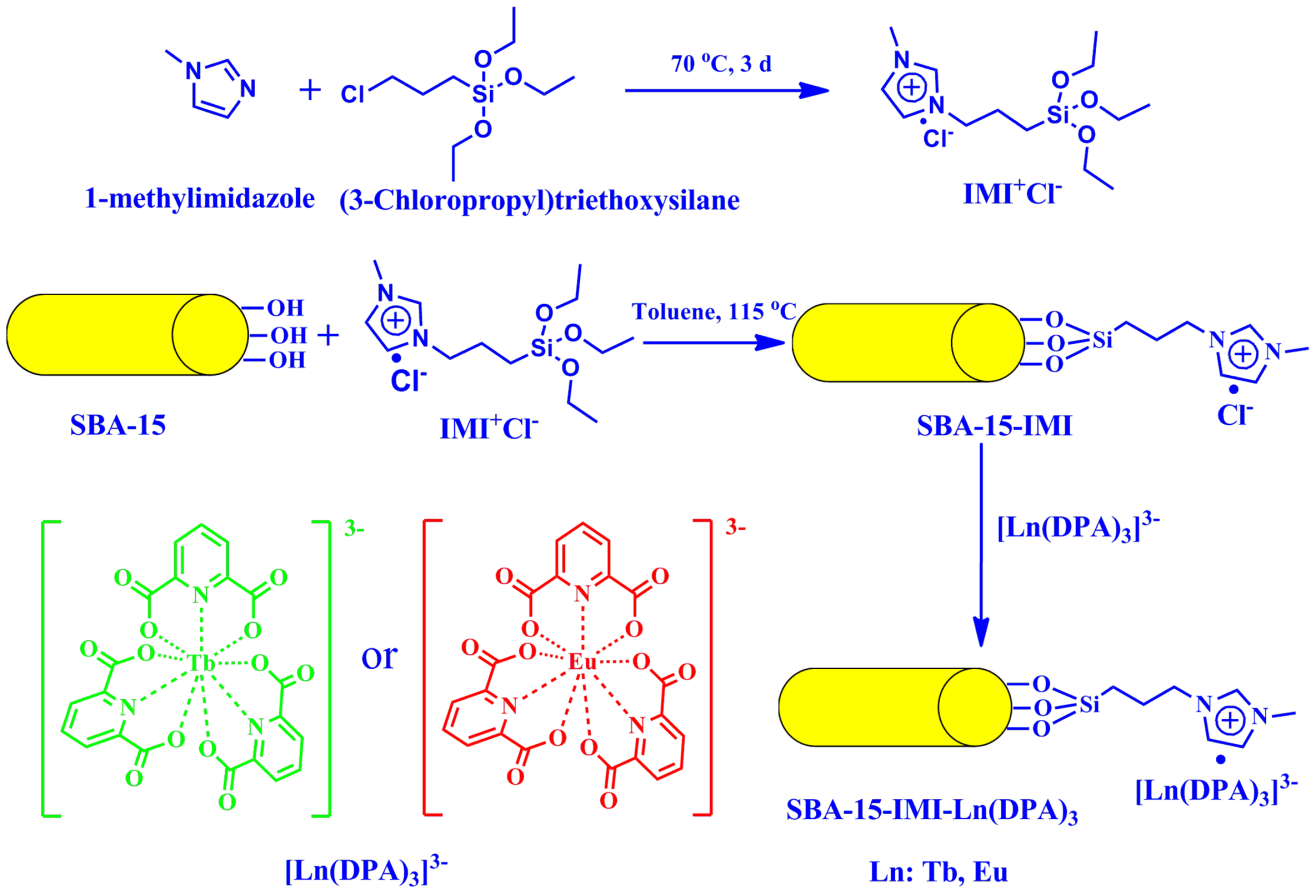
Synthesis procedures of IMI^+^Cl^−^ and the mesoporous luminescence material SBA-15-IMI-Ln(DPA)_3_.

The synthetic scheme of 1-methyl-3-(3-triethoxysilylpropyl) imidazolium chloride (IMI^+^Cl^−^) and the hybrid mesoporous luminescence material (SBA-15-IMI-Ln(DPA)_3_) is shown in Figure 1.

**Figure 2:**
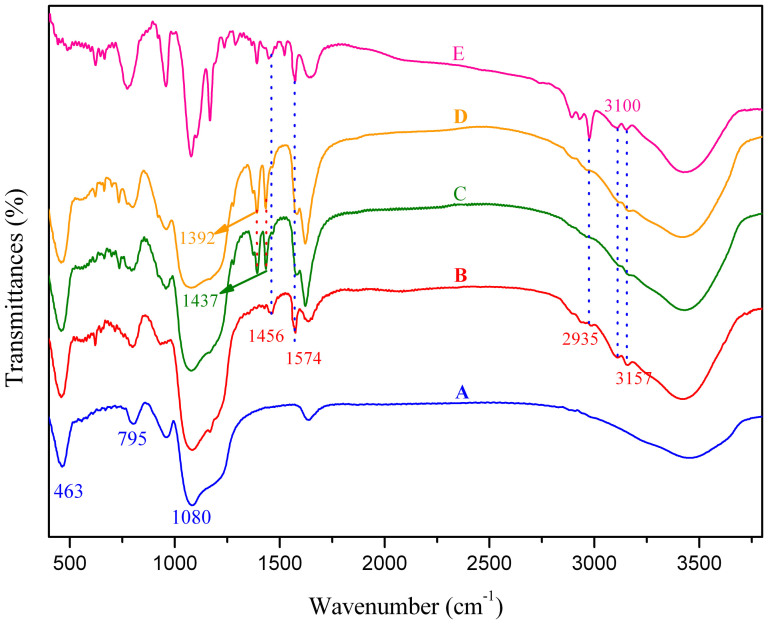
FT-IR spectra of SBA-15 (A), SBA-15-IMI (B), SBA-15-IMI-Tb(DPA)_3_ (C), SBA-15-IMI-Eu(DPA)_3_ (D) and IMI^+^Cl^−^ (E).

Fourier transform infrared (FT-IR) spectrum is commonly used for structural characterization of hybrid materials because it can provide surface information of samples for identification of chemical functional groups. The FT-IR spectra of SBA-15 and functionalized SBA-15 are shown in Figure 2. As shown in Figure 2B and E, the characteristic absorbance for skeleton vibration of imidazolium ring is found at about 1574 and 1456 cm^−1^ 35, meanwhile the bands corresponding to the vibration of saturated C-H and unsaturated C-H appear at 2935 cm^−1^ and 3100, 3157 cm^−1^, respectively, implying that IMI^+^Cl^−^ has been successfully grafted onto the framework of SBA-15^35^. Furthermore, the formation of the Si-O-Si framework is evidenced by the bands located at 1080 cm^−1^ (ν_as_, Si-O), 795 cm^−1^ (ν_s_, Si-O) and 463 cm^−1^(δ, Si-O-Si)^36^, respectively (Figure 2A). The spectra of those lanthanide complex functionalized SBA-15 display two new peaks at 1437 and 1392 cm^−1^, which can be assigned to the asymmetric and symmetric stretch of the carboxylate group, respectively (Figure 2C and D)^37^. This means that the lanthanide DPA complexes have been anchored onto the ionic liquid modified SBA-15 frameworks by anion exchange.

**Figure 3:**
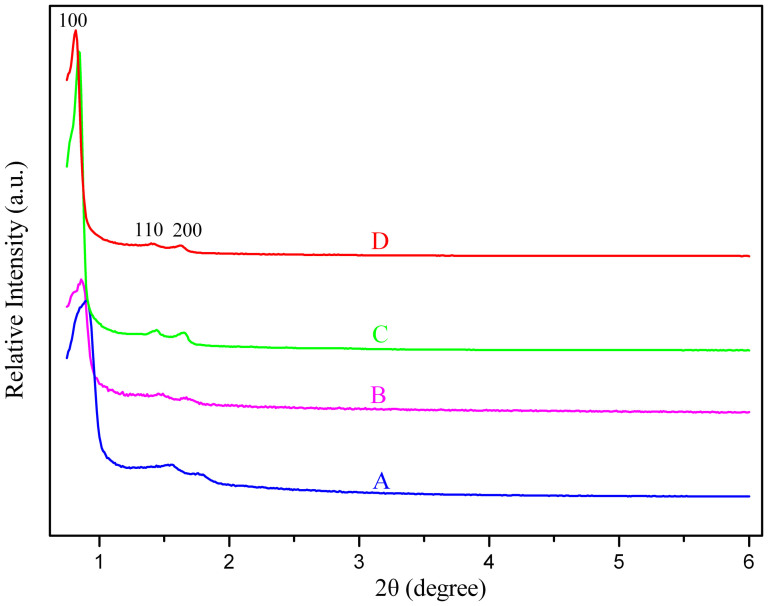
XRD patterns of pure SBA-15 (A), SBA-15-IMI (B), SBA-15-IMI-Tb(DPA)_3_ (C) and SBA-15-IMI-Eu(DPA)_3_ (D).

Figure 3 shows the X-ray powder diffraction (XRD) patterns of pure SBA-15 and modified SBA-15. All the samples clearly display a high-intensity (100) reflection and two weak reflections that can be indexed as the (110) and (200) diffractions respectively, which are characteristic of well-ordered mesoporous material with 2D-hexagonal structure. The hexagonal structures were still clearly observed in the functionalized SBA-15, indicating that the ordered hexagonal mesoporous is maintained during the functionalization of SBA-15 with lanthanide complexes.

**Figure 4:**
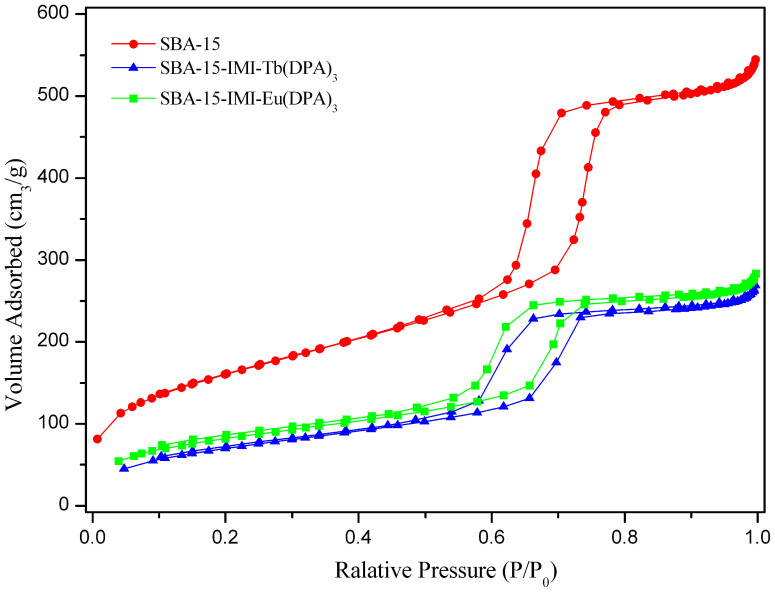
N_2_ adsorption-desorption isotherms of SBA-15, SBA-15-IMI-Tb(DPA)_3_, SBA-15-IMI-Eu(DPA)_3_.

The N_2_ adsorption-desorption isotherms of the SBA-15, SBA-15-IMI-Tb(DPA)_3_ and SBA-15-IMI-Eu(DPA)_3_ are displayed in Figure 4. All of the samples exhibit the type IV adsorption isotherms with H1-type hysteresis loops at high relative pressure, which is a typical characteristics of ordered mesoporous materials^38^. A sharp adsorption step occurs approximately at P/P_0_ = 0.6–0.8, which reveals that those samples possess a well-defined array of regular mesoporous. The results indicated that the mesoporous structure of SBA-15 has been successfully retained under the functionalization environment. The structure data of these mesoporous materials are summarized in Table S1. As expected, the surface area, pore volume and average pore diameter of the modified SBA-15 decrease considerably compared to pure SBA-15, which is consistent with the presence of anchored lanthanide complexes inside the channels of SBA-15^39^.

**Figure 5:**
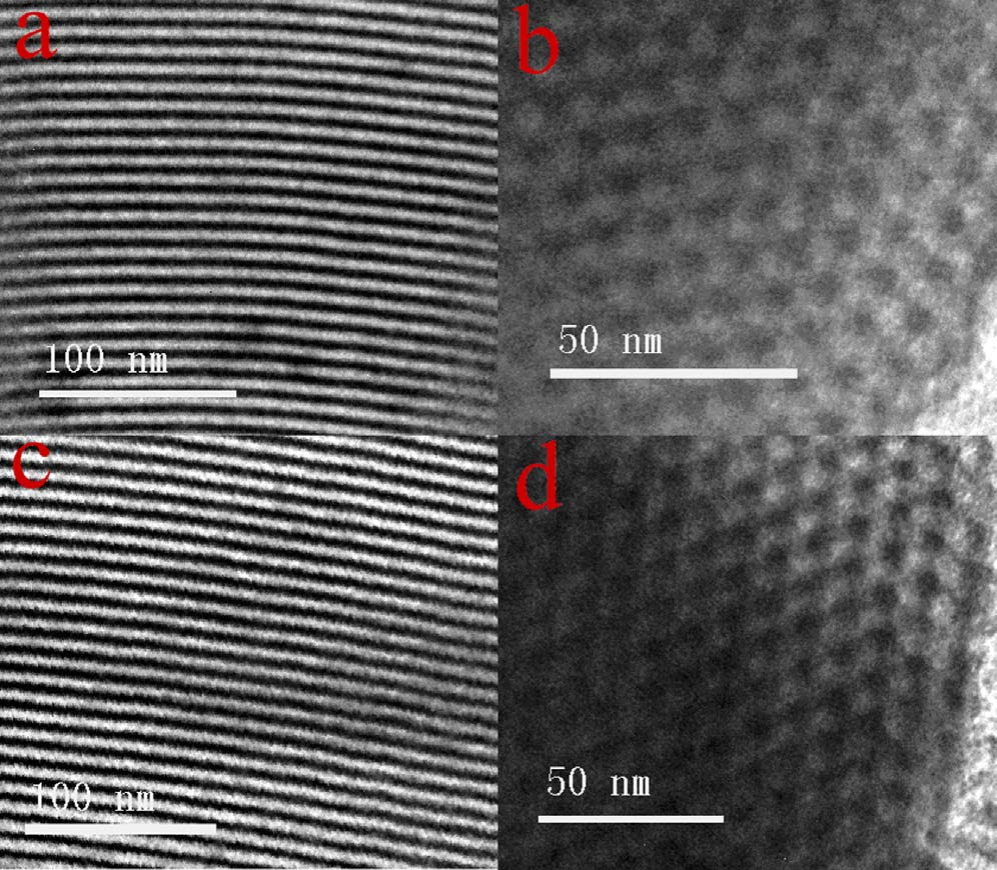
The selected TEM images of hybrid materials SBA-15-IMI-Tb(DPA)_3_ (a, b) and SBA-15-IMI-Eu(DPA)_3_ (c, d).

The morphology and the porous structure features of the mesoporous materials can be observed in Figure S1 and Figure 5. The scanning electron microscopy (SEM) images of SBA-15 and modified SBA-15 display the short rod-shaped external morphology whereas the transmission electron microscopy (TEM) images exhibit a well-ordered 2D hexagonal (P6mm) mesostructure, which is the characteristic structure feature of the SBA-15. This result clearly indicates that the mesoporous structure of SBA-15 is not disrupted after modifying with lanthanide complexes. The energy dispersive X-ray spectroscopy (EDS) analysis indicated that Tb or Eu-element has been detected in SBA-15-IMI-Tb(DPA)_3_ or SBA-15-IMI-Eu(DPA)_3_. (Figure S2).

**Figure 6:**
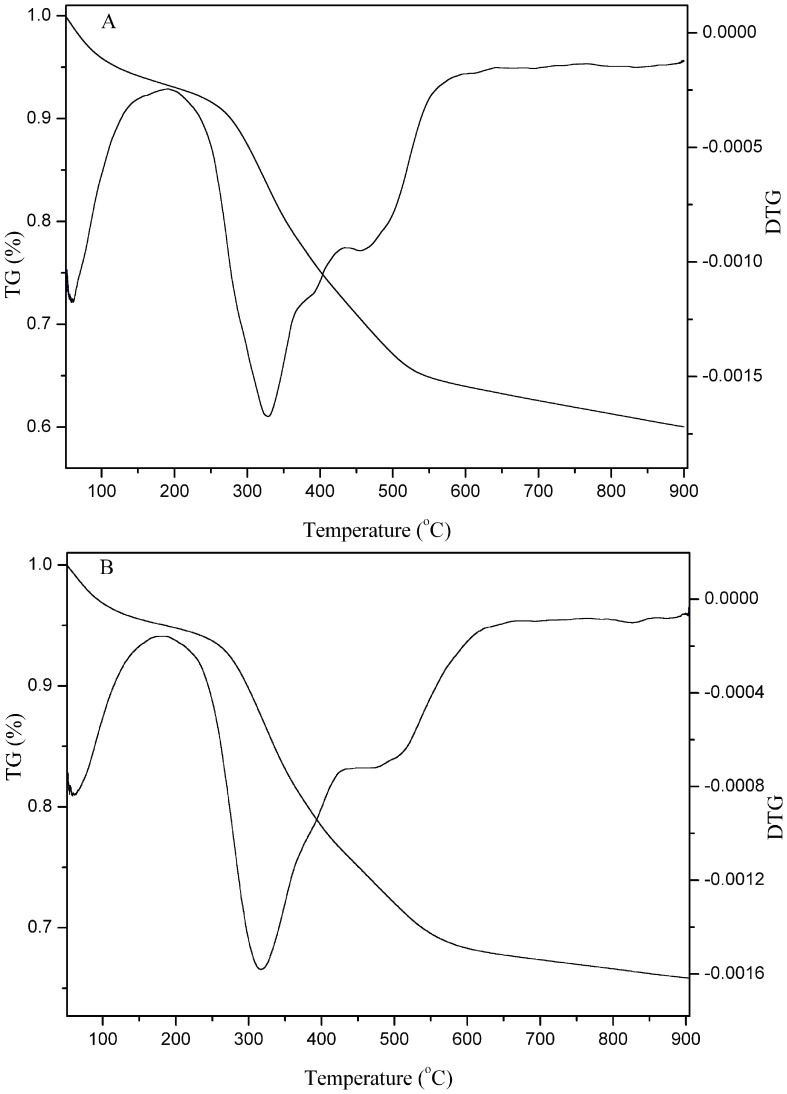
Thermogravimetric (TG) and derivative thermogravimetric (DTG) curves of SBA-15-IMI-Tb(DPA)_3_ (A) and SBA-15-IMI-Eu(DPA)_3_ (B).

Thermogravimetric analysis is one common used thermal analysis technique, which is particularly useful for observing the thermal decomposition of organic-inorganic hybrid materials. The thermogravimetric (TG) curves and the corresponding derivative thermogravimetric (DTG) curves of SBA-15-IMI-Tb(DPA)_3_ and SBA-15-IMI-Eu(DPA)_3_ are shown in Figure 6. The weight loss observed for all the functionalized samples is at two temperatures range. The first important weight loss below 200°C is related to physically adsorbed and coordinated water molecules. The relatively large weight loss occurs between 300 and 550°C, which is assigned to the decomposition of organic species. Beyond this temperature, the weight loss is less obvious. The total weight losses for SBA-15-IMI-Tb(DPA)_3_ and SBA-15-IMI- Eu(DPA)_3_ are about 35.2% and 30.5%, respectively, which indicates that the organic groups have been anchored onto the surface of SBA-15^11^,^40^.

**Figure 7:**
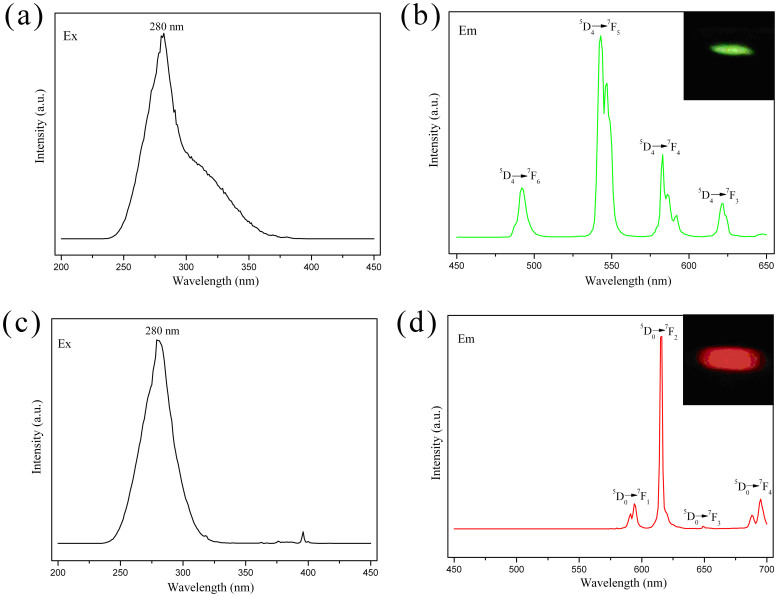
The excitation (a, c) and emission (b, d) spectra of hybrid material SBA-15-IMI-Tb(DPA)_3_ (a, b) and SBA-15-IMI-Eu(DPA)_3_ (c, d), and insets of Figure 7b and 7d are photos of SBA-15-IMI-Tb(DPA)_3_ (green) and SBA-15-IMI-Eu(DPA)_3_ (red) excited by ultraviolet light at 280 nm.

The excitation and emission spectra of SBA-15-IMI-Tb(DPA)_3_ and SBA-15-IMI-Eu(DPA)_3_ are shown in Figure 7. The excitation spectra were recorded by monitoring the ^5^D_4_ → ^7^F_5_ transition at 543 nm for Tb^3+^ and the ^5^D_0_ → ^7^F_2_ transition at 615 nm for Eu^3+^. Both of the spectra display a broad excitation band between 240 nm to 350 nm (λ_max_ = 280 nm), which can be ascribed to the absorption of the complexes. Meanwhile, the maximum absorption of the lanthanide complexes could also be observed at 280 nm in the UV-Vis diffuse reflection spectra (Figure S3). Upon excitation with 280 nm ultraviolet light, the obtained SBA-15-IMI-Tb(DPA)_3_ material gives characteristic emission of Tb^3+^, and the emission peaks at 492, 543, 583 and 622 nm can be assigned to the ^5^D_4_ → ^7^F*_J_* (*J* = 6, 5, 4, 3) transitions, respectively, with the ^5^D_4_ → ^7^F_5_ transition as the most prominent one^41^,^42^. Under the excitation of ultraviolet light with the same wavelength, SBA-15-IMI-Eu(DPA)_3_ reveal the characteristic Eu^3+^ emission peaks centered at 594, 615, 649 and 695 nm, corresponding to the ^5^D_0_ → ^7^F*_J_* (*J* = 1, 2, 3, 4) transitions respectively^43^,^44^. Among these transitions, the ^5^D_0_ → ^7^F_2_ transition (the strongest one) is a typical electric dipole transition which is fairly sensitive to the local symmetry of Eu^3+^ ions, whereas the ^5^D_0_ → ^7^F_1_ corresponds to a magnetic dipole transition that is independent of the host material. According to the transition probabilities, ^5^D_0_ → ^7^F_2_ electron dipole transition becomes the strongest emission when Eu^3+^ is in a noninversion symmetry site, while in a center with inversion, the ^5^D_0_ → ^7^F_1_ magnetic dipole transition is dominant^45^. Therefore, the relative intensity ratio (R_I_) of ^5^D_0_ → ^7^F_2_ (red light) to ^5^D_0_ → ^7^F_1_ (orange red light) depends strongly on the local symmetry around the Eu^3+^ ion. The value of R_I_ for SBA-IMI-Eu(DPA)_3_ is 7.6, suggesting that the local surrounding of Eu^3+^ is highly asymmetric without an inversion center. In addition, the full width at half maximum of the strongest band is less than 4 nm, which shows that the SBA-15-IMI-Eu(DPA)_3_ exhibits a high color purity. As observed by the luminescence photos (Figure 7 b and d, insets), the hybrid materials SBA-15-IMI-Tb(DPA)_3_ and SBA-15-IMI-Eu(DPA)_3_ exhibit bright green and red color luminescence respectively upon irradiation with a single wavelength (280 nm) ultraviolet light.

The chromaticity diagram (CIE) shown in Figure S4 indicates that the emission of both hybrids lie in green and red region with the chromaticity coordinates (*x* = 0.35, *y* = 0.59) for SBA-15-IMI-Tb(DPA)_3_ and (*x* = 0.66, *y* = 0.33) for SBA-15-IMI-Eu(DPA)_3_, respectively. Moreover, the chromaticity coordinate of the SBA-15-IMI-Eu(DPA)_3_ is close to the edge of the CIE diagram, which also shows this sample with high color purity.

**Figure 8:**
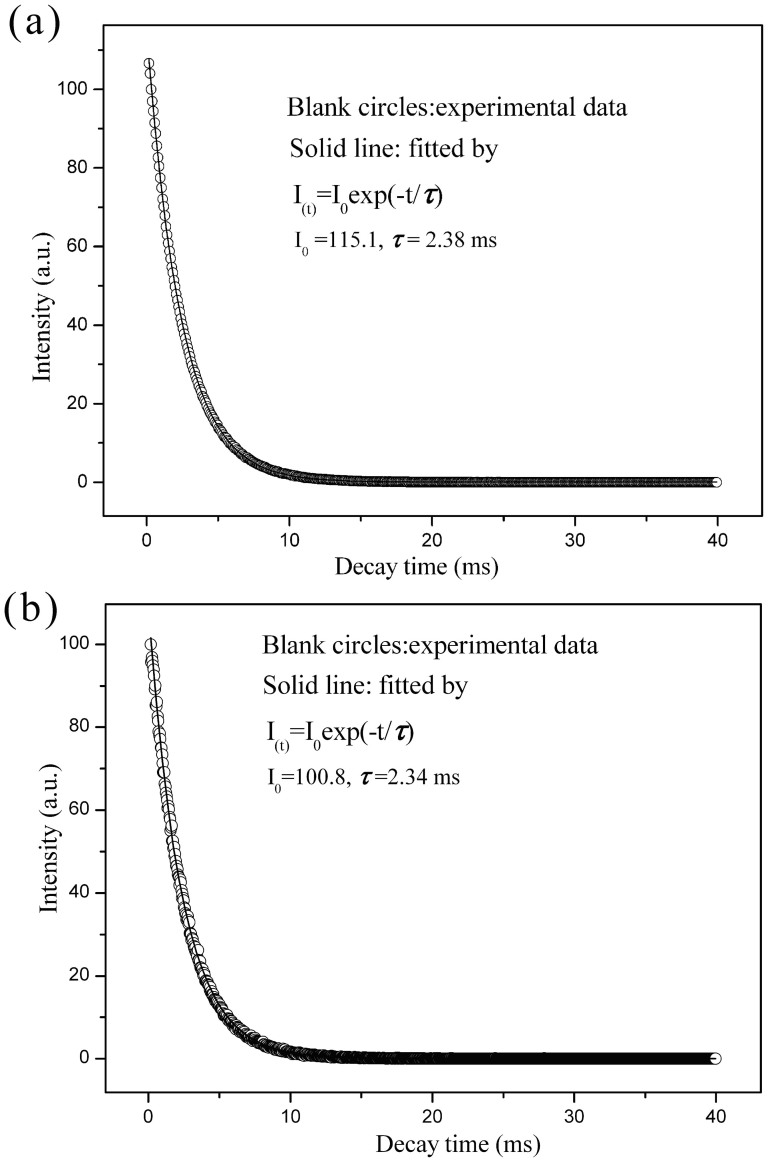
Luminescence decay curves of the hybrid materials, SBA-15-IMI-Tb(DPA)_3_ (a), SBA-15-IMI-Eu(DPA)_3_ (b).

The luminescence decay curves of SBA-15-IMI-Tb(DPA)_3_ and SBA-15-IMI-Eu(DPA)_3_ are shown in Figure 8. Both of the curves can be well fitted by a single exponential function described as equation (1)
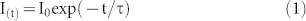
Where τ stands for the luminescence lifetime and I_0_ is the corresponding fitting parameter. The values of τ are determined to be 2.38 ms for SBA-IMI-Tb(DPA)_3_ and 2.34 ms for SBA-IMI-Eu(DPA)_3_, respectively. The results indicate that the average chemical environments of Tb^3+^ or Eu^3+^ are uniform. The luminescence lifetime of lanthanides is greatly affected by the vibration of the nearby hydroxyl groups and ligands. The vibrations of hydroxyl groups and ligands will absorb the excitation energy of Tb^3+^ or Eu^3+^, leading to decrease the lifetime of the lanthanides. As for our hybrid luminescence materials, the strong electrostatic interaction between the matrix and the lanthanide complexes limits the vibration of the ligands around lanthanides. Furthermore, [Ln(DPA)_3_]^3−^ have no coordinated water molecules, leading to long luminescence lifetimes of lanthanides. The quantum efficiencies (η) of the SBA-15-IMI-Tb(DPA)_3_ and SBA-15-IMI-Eu(DPA)_3_ are about 63% and 79%, respectively. The photoluminescence data including excitation wavelength, emission wavelength, quantum efficiency and lifetime of our samples and other analogous materials are listed in Table 1, it appears that the lifetime in SBA-15-IMI-Ln(DPA)_3_ (Ln = Tb, Eu) is longer than that in other hybrid materials containing a similar lanthanide complexes. The high quantum efficiencies and long luminescence lifetimes suggest that this method is a preferable way for constructing hybrid mesoporous materials with bright green or red emission.

**Figure 9:**
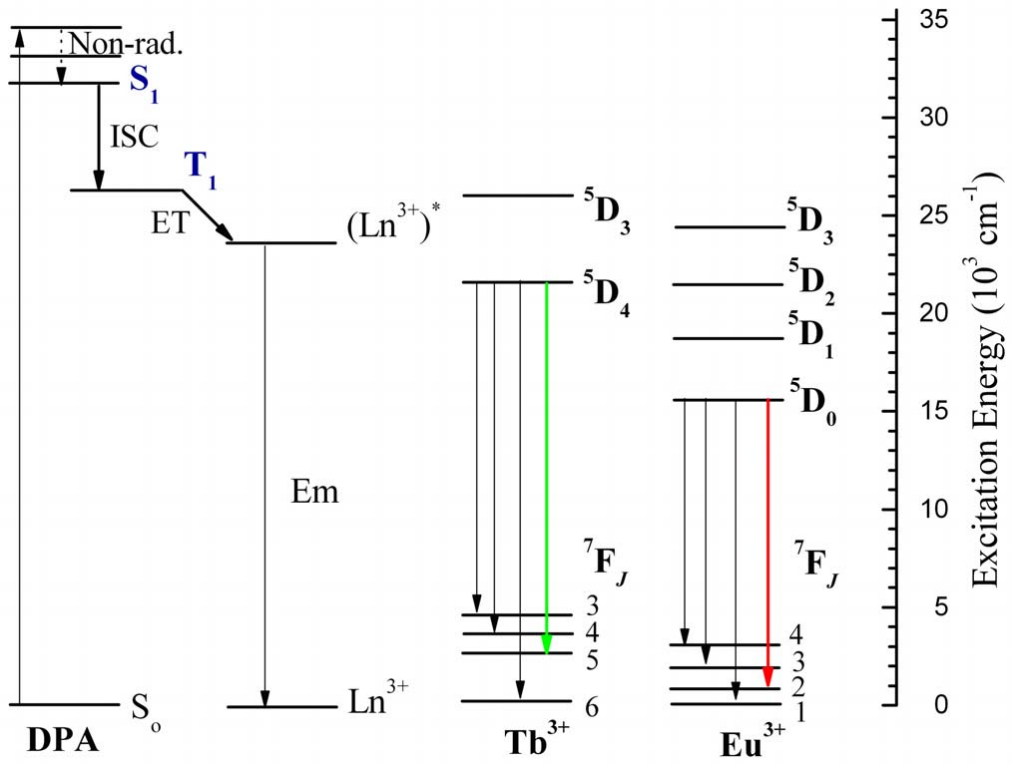
Simplified energy level and electron transition diagram of [Ln(DPA)_3_]^3−^ system. Non-rad = nonradiative transitions, ISC = intersystem crossing, ET = energy transfer, S = Singlet, T = triplet.

The reason that the excellent luminescent properties of SBA-15-IMI-Ln(DPA)_3_ is mainly attributed to two aspects. On the one hand, As can be seen in Figure S5, three DPA ligands are able to coordinate with the Ln^3+^ through six oxygen atoms of carboxyl groups and three nitrogen atoms of pyridine units, and the coordination sphere of Ln^3+^ is saturated by the ligands, leaving no coordination site for water and other solvent molecules. On the other hand, the energy levels between DPA ligand and Ln^3+^ are well matched and there exist efficient energy transfer between them. It has been confirmed that the matching degree between the lowest triplet state energy level of ligand and emission energy level of Ln^3+^ is a critical factor which affects the luminescence properties of the lanthanide complexes^47^. Generally, the energy transfer between the organic ligand and the center lanthanide may be proceeded when the lowest energy of the triplet state of the ligands is higher than the energy of the resonance levels of the lanthanides. For the DPA ligand, the lowest energy of the triplet state of the DPA (26600 cm^−1^)^30^ is higher than the resonant energy level of Tb^3+^ (^5^D_4_, 21500 cm^−1^) and Eu^3+^ (^5^D_0_, 17250 cm^−1^), indicating that DPA would be an efficient ligand for sensitizing the luminescence of Tb^3+^ and Eu^3+^. The simplified energy levels, electron transition schematic diagram and the luminescent mechanism are illustrated in Figure 9. First, the DPA complex is excited from the singlet ground state (S_0_) to excited singlet states by absorbing the light energy, and then relaxed to the lowest excited singlet states (S_1_) through non-radiative transitions. Secondly, the energy of excited singlet state (S_1_) of the ligand is transferred to its excited triplet state (T_1_) through intersytem crossing, Thirdly, the intramolecular energy transfer from the excited triplet state of the ligands to the excited 4f states of the Tb^3+^ ions (^5^D_4_) or Eu^3+^ ions (^5^D_0_) through non-radiative transition. Finally, the radiative transition of Ln^3+^ produces the characteristic luminescence of Ln^3+^.

## Conclusions

In this work, two novel luminescence mesoporous hybrid materials have been prepared by incorporating [Ln(DPA)_3_]^3−^ into ionic liquid modified SBA-15 through strong electrostatic interactions. The mesoporous structures of SBA-15 can be retained after functionalization with [Ln(DPA)_3_]^3−^ (Ln: Tb, Eu). Under the excitation of ultraviolet light with the same wavelength (280 nm), the hybrid materials show the characteristic emission of Tb^3+^ (green) and Eu^3+^ (red) with long luminescence lifetimes and high quantum efficiencies, which indicates that [Ln(DPA)_3_]^3−^ are very suitable as the luminescence center for construction of the hybrid luminescence materials.

## Methods

### Synthesis of 1-methyl-3-(3-triethoxysilylpropyl) imidazolium chloride (IMI^+^Cl^−^)

1-methyl-3-(3-triethoxysilylpropyl) imidazolium chloride was synthesized from (3-Chloropropyl)triethoxysilane and 1-methylimidazole as previously reported^48^.

### Synthesis of mesoporous material SBA-15^49^

Poly(ethylene oxide)-poly(propylene oxide)-poly(ethylene oxide) triblock copolymer (Pluronic P123, EO_20_PO_70_EO_20_, *M*_av_ = 5800, 4.0 g) was dissolved in hydrochloric acid (120 mL, 2 mol/L) and distilled water (30 mL) under stirring. Then tetraethoxysilane (TEOS, 8.4 g) was added dropwise to the above mixture solution under vigorous stirring at 313 K. After TEOS was completely added, the mixture was left to stirred at 313 K for 24 h and then transferred into a hermetically sealed Erlenmeyer flask, and kept at 353 K for 48 h. Finally the product was recovered by filtration, washed with distilled water and air-dried. The template was removed by Soxhlet extraction with hydrochloric acid and ethanol solution under reflux for 2 d.

### Synthesis of hybrid mesoporous material SBA-15-IMI^50^

1-methyl-3-(3-triethoxysilylpropyl) imidazolium chloride (IMI^+^Cl^−^, 3 mL) and dry triethylamine (3 mL) were added dropwise to a suspension of SBA-15 (2 g) in dry toluene (60 mL). The resulting mixture was refluxed at 115°C for 24 h under an atmosphere of argon. After cooling, the suspension was centrifuged and the adsorptive ionic liquid was removed by Soxhlet extraction with dry dichloromethane under reflux for 24 h. The resulting powder, denoted as SBA-15-IMI, was dried under vacuum at 60°C for 10 h.

### Synthesis of hybrid mesoporous luminescence material SBA-15-IMI-Ln(DPA)_3_

Pyridine-2, 6-dicarboxylic acid (DPA, 1.5 mmol) was added to an aqueous solution of Tb(NO_3_)·6H_2_O or Eu(NO_3_)·6H_2_O (0.5 mmol, 20 mL), and the pH was adjusted to be neutral by adding NaHCO_3_ (According to the above procedure, the crystals of the lanthanide complexes (Na_3_[Ln(DPA)_3_], Ln = Tb, Eu) were obtained by the solvent evaporation method). The mixture was left to stir for 2 h, and then SBA-15-IMI (0.5 g) was added to the above solution. After stirred at room temperature for about 24 h, the resulting product, denoted as SBA-15-IMI-Tb(DPA)_3_ or SBA-15-IMI-Eu(DPA)_3_, was filtered out and washed with water, air-dried.

Fourier transform infrared (FT-IR) spectrum was measured using a Nicolet 5700 infrared spectrometer by the KBr tablet method. X-ray powder diffraction patterns were recorded on Bruker D8 FOCUS diffractometer using Cu target (voltage: 40 kV, current: 40 mA) equipped with a scintillation crystal detector. Single crystal X-ray diffraction data were collected on a Bruker SMART APEX II diffractometer. Data reductions and absorption corrections were performed with the SAINT and SADABS programs, respectively. The structures were solved by direct methods and refined with full-matrix least squares on *F*^2^ using the SHELXS-97 program. N_2_ adsorption-desorption isotherms were obtained on a Micromeritics Tristar Π 3020 sorptometer. The pore size distribution calculated from adsorption isotherms based on the Barrett-Joyner-Halenda (BJH) method. The structure and composition of the samples were inspected using a JEOL-2100F transmission electron microscope (TEM) equipped with an energy-dispersive X-ray spectroscopy (EDS) with an accelerating voltage of 200 kV. The scanning electron microscopy (SEM) was obtained from a FEI Quanta 200 scanning electron microscope. Thermogravimetric (TG) analysis was performed on a STA 6000 (Perkin Elmer) instrument. Ultraviolet-Visible (UV-Vis) diffused reflection spectra measurements were carried out on a TU-1901 (Purkinje) UV-Vis spectrophotometer. Photoluminescence (PL) spectrum and luminescence lifetime measurements were carried out on an FLS920P Edinburgh instruments apparatus equipped with a 450 W xenon lamp and a high-energy micro-second flash lamp as the excitation sources. The quantum efficiencies were determined using an integrating sphere whose inner face was coated with BenFlect from FLS920P Edinburgh spectrofluorometer.

## Author Contributions

L.Q.F. and W.Z.L. performed the experiments and wrote the paper. Y.D. prepared all the figures. L.W., Z.X.L. and L.C.Y. carried out the materials characterization. All authors discussed the experimental results and reviewed the manuscript.

## Supplementary Material

Supplementary Information Hybrid luminescence materials assembled by [Ln(DPA)3]3- and mesoporous host through ion-pairing interactions with high quantum efficiencies and long lifetimes

## Figures and Tables

**Table 1:** Photoluminescence data of the SBA-15-IMI-Ln(DPA)_3_, (Ln = Tb, Eu) and previously reported hybrid luminescence mesoporpous materials. excitation wavelength (λ (ex)), emission wavelength (λ (em)), lifetime (τ), quantum efficiency (η), Reference (ref)

Samples	λ (ex)	λ (em)	τ (ms)	η (%)
SBA-15-IMI-Tb(DPA)_3_	280 nm	492, 543, 583, 622 nm	2.38	63
SBA-15-IMI-Eu(DPA)_3_	280 nm	594, 616, 649, 695 nm	2.34	79
Tb-dpa-Si-MSNs *^Ref. ^8*	281 nm	490, 546, 582, 622 nm	0.86	
Eu-dpa-Si-MSNs *^Ref. ^8*	281 nm	592, 614, 650, 694 nm	0.62	
Eu(PEG-SBA-15)_3 _*^Ref. ^6*	349 nm	590, 611, 650, 700 nm	0.943	37.77
Eu(PEG-SBA-15)_3_phen *^Ref. ^6*	351 nm	590, 611, 650, 700 nm	0.952	71.11
4a *^Ref. ^46*	350 nm		0.596	12.81
