# Supplementary material for: Hybrid luminescence materials assembled by [Ln(DPA)3]3− and mesoporous host through ion-pairing interactions with high quantum efficiencies and long lifetimes
Source: Sci Rep. 2015 Feb 11;5:8385. doi: 10.1038/srep08385 (PMC4323642; doi:10.1038/srep08385)
Supplement: Supplementary Information — Hybrid luminescence materials assembled by [Ln(DPA)3]3- and mesoporous host through ion-pairing interactions with high quantum efficiencies and long lifetimes [file srep08385-s1.doc]

**Supplementary Information**

**Hybrid luminescence materials assembled by [Ln(DPA)_3_]^3-^ and mesoporous host through ion-pairing interactions with high quantum efficiencies and long lifetimes**

Qing-Feng Li,^a^* Dan Yue, ^a^ Wei Lu,^b^ Xinlei Zhang,^a^ Chunyang Li,^a^ Zhenling Wang^a^*

*^a^*The Key Laboratory of Rare Earth Functional Materials and Applications, Zhoukou Normal University, Zhoukou 466001, P. R. China.

*^b^*Department of Applied Physics and Materials Research Center, The Hong Kong Polytechnic University, Hong Kong, P. R. China

*Corresponding authors. Tel.: +86-394-8178518; fax: +86-394-8178518.

*E-mail address*: liqingf335@163.com (Q. Li); zlwang2007@hotmail.com (Z. Wang)

**Table S1** Structural parameters for hybrid mesoporous silicas, Brunauer-Emmett-Teller (BET) surface area (S), total pore volume (V), average pore diameter (D)

| Sample | S (m^2^ g^-1^) | V (cm^3^ g^-1^) | D (nm) |
| --- | --- | --- | --- |
| SBA-15 | 570 | 0.85 | 8.43 |
| SBA-15-IMI-Tb(DPA)_3_ | 259 | 0.44 | 7.65 |
| SBA-15-IMI-Eu(DPA)_3_ | 279 | 0.45 | 7.25 |


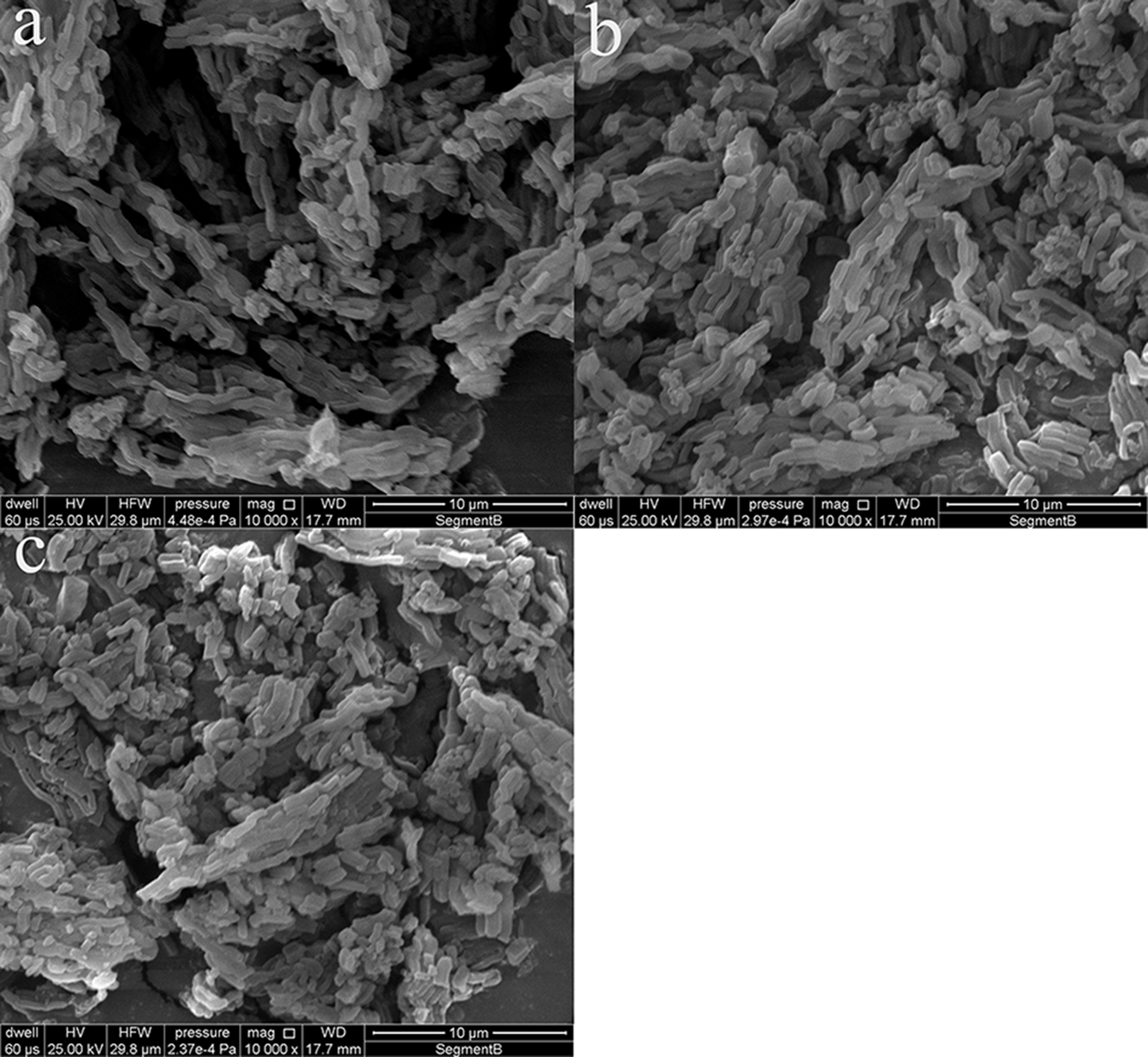


**Figure S1:** SEM images of SBA-15 (a), SBA-15-IMI-Tb(DPA)_3_ (b), SBA-15-IMI-Eu(DPA)_3_ (c)


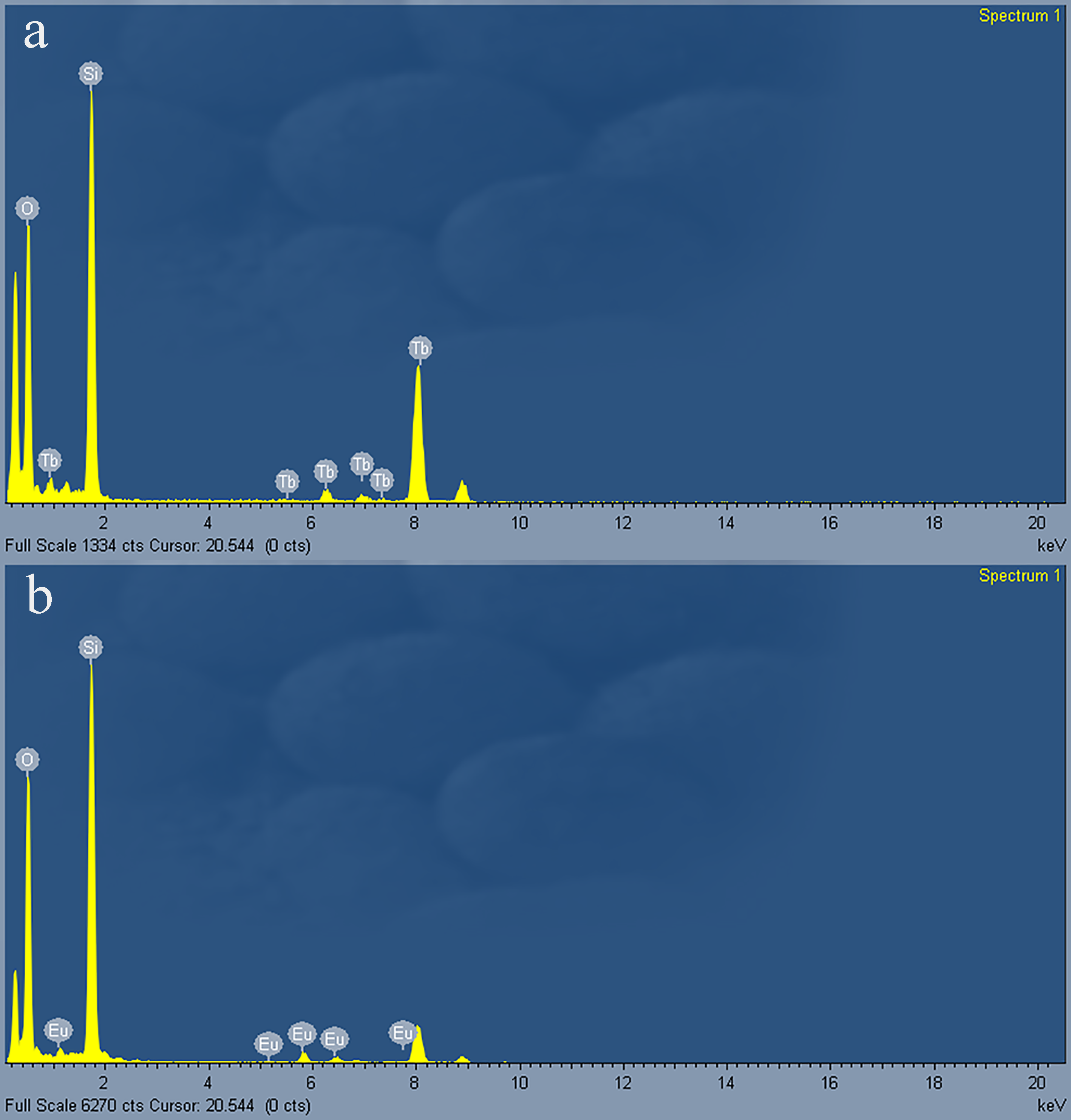


**Figure S2:** EDS graphs of SBA-15-IMI-Tb(DPA)_3_ (a), SBA-15-IMI-Eu(DPA)_3_ (b)


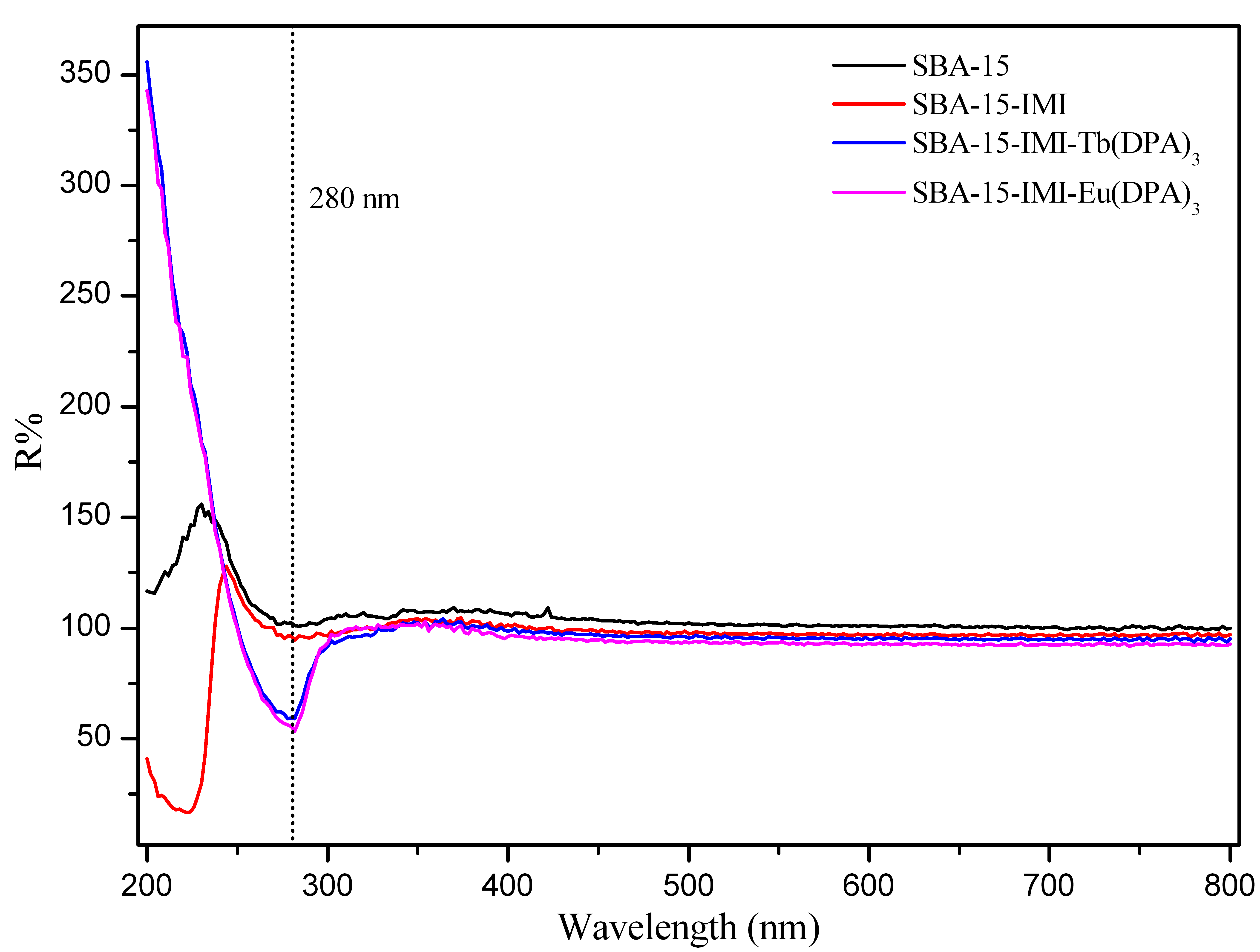


**Figure S3**: UV-Vis diffuse reflection spectra of SBA-15 and modified SBA-15


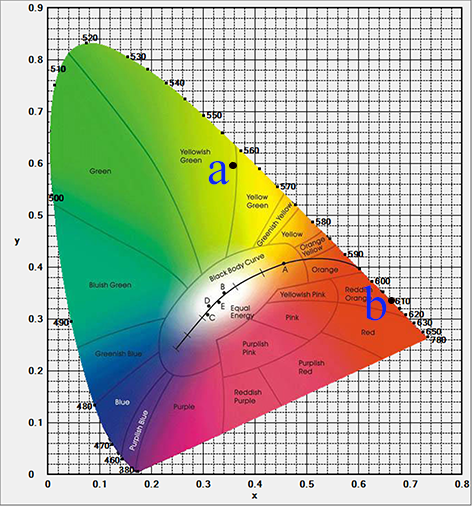


**Figure S4**: Chromaticity diagram (CIE) of the SBA-15-IMI-Tb(DPA)_3_ (a) and SBA-15-IMI-Eu(DPA)_3_ (b)


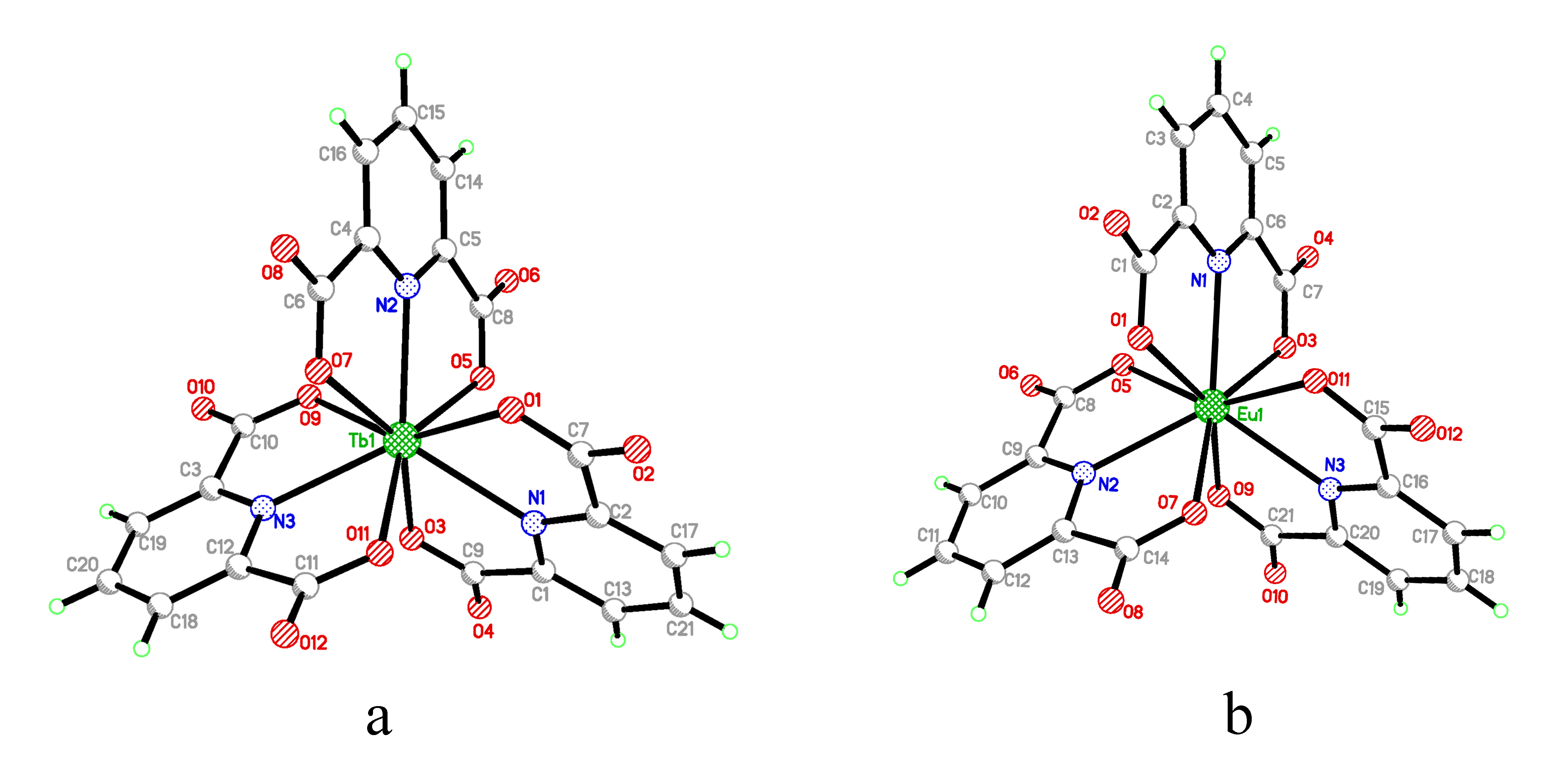


**Figure S5:** The crystal structures of lanthanide complexes, Na_3_[Tb(DPA)_3_] (a) and Na_3_[Eu(DPA)_3_] (b), DPA: 2,6-Pyridinedicarboxylic acid
